# Assessing Error Awareness as a Mediator of the Relationship between Subjective Concerns and Cognitive Performance in Older Adults

**DOI:** 10.1371/journal.pone.0166315

**Published:** 2016-11-10

**Authors:** Rachel F. Buckley, Gemma Laming, Li Peng Evelyn Chen, Alice Crole, Robert Hester

**Affiliations:** 1 Florey Institute of Neuroscience and Mental Health, Melbourne, Australia; 2 Melbourne School of Psychological Sciences, University of Melbourne, Melbourne, Australia; 3 Department of Neurology, Harvard Medical School and Massachusetts General Hospital, Boston, Massachusetts, United States of America; Center for BrainHealth, University of Texas at Dallas, UNITED STATES

## Abstract

**Objectives:**

Subjective concerns of cognitive decline (SCD) often manifest in older adults who exhibit objectively normal cognitive functioning. This subjective-objective discrepancy is counter-intuitive when mounting evidence suggests that subjective concerns relate to future clinical progression to Alzheimer’s disease, and so possess the potential to be a sensitive early behavioural marker of disease. In the current study, we aimed to determine whether individual variability in conscious awareness of errors in daily life might mediate this subjective-objective relationship.

**Methods:**

67 cognitively-normal older adults underwent cognitive, SCD and mood tests, and an error awareness task.

**Results:**

Poorer error awareness was not found to mediate a relationship between SCD and objective performance. Furthermore, non-clinical levels of depressive symptomatology were a primary driving factor of SCD and error awareness, and significantly mediated a relationship between the two.

**Discussion:**

We were unable to show that poorer error awareness mediates SCD and cognitive performance in older adults. Our study does suggest, however, that underlying depressive symptoms influence both poorer error awareness and greater SCD severity. Error awareness is thus not recommended as a proxy for SCD, as reduced levels of error awareness do not seem to be reflected by greater SCD.

## Introduction

Subjective concerns of cognitive decline (SCD) are common in healthy older adults, and growing evidence suggests that SCD predicts future progression to AD [1]. Despite this, evidence is equivocal on whether SCD is indicative of objective cognitive decline; some studies report small to moderate effects [2], while others have failed to find a relationship [3]. This has led some researchers to question the clinical utility of SCD at the earliest stages of the disease [4], and what variables influence the discrepancy between subjective and objective cognitive performance.

One rationale for the inaccuracy of SCD is via the influence of individual variability in error awareness, which is exacerbated by age-related changes [5, 6]. Error detection is a vital component of adaptive human behaviour, which allows learning to be efficient and reflexive to the environment [7]. The awareness of making an error forms the foundation of metacognitive monitoring of cognitive function, and allows for subsequent implementation of adaptive cognitive processes. Poorer error awareness during cognitive task performance in older adults has been found to relate to poorer general self-awareness [5], leading to the implication that individual differences in error awareness may well impinge on negative self-appraisals of cognitive performance.

Evidence from cognitive experimental literature suggests that error-related negativity (ERN), an electrophysiological marker of error detection, aids in the development of adaptive behaviours, while error-related positivity (ERP) was found to be indicative of error awareness [8]. Functional magnetic resonance imaging (fMRI) studies implicate the dorsal anterior cingulate cortex (dACC), insular and bilateral inferior parietal cortices for error detection [9–11], while error awareness has been associated with BOLD response in the prefrontal cortex, parietal cortex, and anterior insular in younger adults [9, 10, 12]. Both error-related responses have been shown to become attenuated in older adults [13–15], with the implication that age-related deterioration of reinforcement-learning signals from dopamine-saturated striatal structures may jeopardise the ability of older adults to attend to and learn from error events [14, 16].

The aim of the current study was to determine if individual differences in conscious error awareness was associated with SCD, and whether error awareness mediated a relationship between SCD and objective cognitive performance. It was hypothesized that poorer monitoring of errors in older adults would contribute to greater SCD severity and would provide a logical mediating link between SCD and objective cognitive performance.

## Materials and Methods

### Participants

Seventy healthy older participants (36 female) were invited to participate in the current study via media appeal to local community newsletters (i.e. Probus, University of the Third Age), and also via an appeal to participants from an online longitudinal healthy ageing study, the Trajectory-Related Early Alzheimer’s Database (TREAD) study [17]. The institutional ethics committee of the University of Melbourne approved this study, and all volunteers gave written informed consent before participating. Participants were screened for current or previous neurological and psychiatric disorders, such as depression, anxiety, epilepsy, stroke, etc. One participant was excluded prior to the assessment on the basis of a current diagnosis of depression, while another was excluded due to poor vision. Two participants performed below 3 standard deviations on the Error Awareness Task (EAT) and were not included in further analyses, thus reducing the total participant group to 67 (32 female).

Individuals who volunteered to take part were excluded if they had a current or past history of psychiatric or neurological illness, significant current depression (Geriatric Depression Score (GDS) above 11/30), a Mini-Mental State Examination (MMSE) score below 24, cancer within the last two years, symptomatic stroke, uncontrolled diabetes, or excessive alcohol use (following previous standards set by the Australian Imaging Biomarker and Lifestyle (AIBL) study of ageing [18].

### Measures

#### Error awareness

The Error Awareness Task (EAT) is a Go/No-Go response inhibition task in which a participant makes errors of commission of which they are aware or unaware [9]. The task involves a serial stream of colour words that are presented in an incongruent colour. The words are presented for approximately 1000ms followed by a 500ms inter-stimulus interval. Participants were required to respond with a button press ‘1’ when the colour of the word and the word were incongruent (Go Trial), but withhold a response when the colour and word were congruent (No-Go trial). If the individual consciously detected a self-made error, they were trained to indicate this in the next trial by pressing ‘2’, and were not required to make the standard Go response.

Six blocks of 175 trials were administered; 25 No-Go trials were distributed pseudo-randomly through the serial presentation of 150 Go trials within each block. The EAT task was programmed to vary stimulus presentation speed between 500ms-1500ms in order to maintain 60% accuracy in overall task performance. All participants were found to hit the maximum stimulus presentation speed. To ensure that speed was not being traded off significantly for accuracy, we ran a correlation analysis, and found no significant association between the two indices, r(67) = 0.07, *p* = .58. The error awareness outcome measure was calculated via a ratio of aware and non-aware errors of commission. Task accuracy and error awareness levels were not related in this study, r(67) = 0.13, *p* = .31. To ensure that there would be no confound, accuracy for each individual was regressed onto their error awareness in order to measure unique individual variability in error awareness beyond age-related task performance [19, 20].

#### Subjective cognitive decline

The Everyday Cognition (ECog) scale has been described in detail elsewhere [21]. Participants were asked to determine whether changes have occurred in the following cognitive domains in the past ten years: memory, visuospatial abilities, language, planning, organizational abilities, and divided attention. Raw scores from the ECog were converted into z-scores for each participant, with higher scores indicating greater SCD. A total SCD composite was created by averaging all z-scores together to create composite [21].

#### Cognitive measures

The CANTABeclipse v3.0 Paired Associate Learning (PAL) task was used to measure non-verbal associative learning, a form of episodic memory. The following norm-adjusted measures from the PAL were used: first trial memory score, mean errors to completion, total number of trials, and total number of errors. Age-adjusted time to complete Trails B (in seconds) was used to measure task-switching ability, and No-Go trial accuracy in the EAT was used to measure response inhibition; both represented components of executive functioning. To create composite cognitive measures, all indices were z-transformed and then averaged to form their respective cognitive composites of episodic memory and executive functioning. A global cognitive composite was also created by averaging all indices together. Higher scores on each composite indicated better performance. Premorbid IQ was estimated from the National Adult Reading Test (NART; [22]).

### Statistical Methods

All analyses were conducted using SPSS software (version 23.0). A series of t-tests and Pearson correlations were conducted to determine whether error awareness or SCD were influenced by demographics variables. These variables were then included as nuisance covariates. In order to determine whether error awareness influenced SCD directly, a hierarchical regression analysis was conducted, accounting for nuisance covariates in the first block and the error awareness residual in the second, and SCD as the dependent variable. The R^2^_change_ score was used to determine whether error awareness contributed significantly to the model over and above the covariates (age, education, GDS score, episodic memory and executive function performance). Finally, bootstrapped mediation analyses were run to determine whether SCD indirectly related to EM or EF performance via the error awareness residual. Our prediction with these models was that error awareness may explain an indirect relationship between SCD and cognitive performance, such that better error awareness would strengthen the aforementioned relationship. We conducted these analyses using the *PROCESS* package developed for SPSS, with SCD as the outcome variable, the cognitive composite as the predictor variable, and the error awareness residual as the mediator [23]. Findings were corrected for multiple comparisons using Sidak adjustment within SPSS.

## Results

### Characteristics of the Sample and Level of Error Awareness

The demographic, affective, cognitive and SCD parameters for the healthy older participants are presented in Table 1. Participants were normally distributed around an average age of 69.4 (*SD* = 5.3), had attained at least a Bachelor’s degree, and exhibited a high FSIQ. Cognitive performance was found to be within age-related norms, and SCD composite scores were normally distributed around the mean.

**Table 1:** Demographic, affective, cognitive and SCD parameters.

	*Mean/Frequency*	*SD*
**Demographics**		
Age (years)	69.4	5.3
Sex (%F)	48	
Education (years)	15.6	3.6
FSIQ	120	6.0
MMSE	29.5	0.9
**Mood**		
GDS	3.07	2.6
**Memory**		
PAL total errors^a^	0.32	0.7
**Executive function**		
Go trial response time (ms)	603.47	58.8
No-Go trial accuracy (%)	73	12
Error awareness (%)	57	29
**SCD measure**		
ECog Memory (range: 8–24)	12.0	5.1
ECog Language (range: 9–27)	12.6	3.7
ECog Visuospatial (range: 8–14)	8.6	1.9
ECog Planning (range: 5–8)	5.5	0.9
ECog Organisational (range: 5–12)	7.1	1.6
ECog Divided Attention (range: 4–11)	5.7	1.9

^a^ age-adjusted score using CANTAB norms.

FSIQ = Full-Scale Intelligence Quotient, MMSE = Mini-Mental State Examination, GDS = Geriatric Depression Scale, PAL = Paired associates learning, ECog = Everyday Cognition questionnaire

Accuracy on the EAT task was found to be significantly higher than the 60% accuracy level set for the task, t(66) = 8.95, *p* < .001. It is possible that participants benefited from the maximum ~1500ms latency period, thus leading to a greater accuracy level. This did not affect level of error awareness, which was found to distribute normally around the mean of 57% (*SD* = 29%).

### Demographic Associations with Error Awareness and SCD Composite

Better error awareness related to increasing depressive symptomatology, r(65) = 0.28, *p* = .02 (see S1 Fig for the scatterplot), but not age, r(66) = 0.02, *p* = .89, FSIQ, r(63) = -0.06, *p* = .65, MMSE score, r(66) = -0.02, *p* = .89, episodic memory, r(65) = 0.02, *p* = .88, executive function, r(67) = -0.07, *p* = .56, or global cognition, r(66) = -0.02, *p* = .89. There was no group effect of gender, t(66) = -0.01, *p* = .99, on error awareness.

Greater SCD also related to increased depressive symptoms, r(66) = 0.46, *p* < .001 (see S2 Fig for the scatterplot), but no association was found with age, r(67) = 0.09, *p* = .47, level of education, r(67) = 0.04, *p* = .75, MMSE, r(67) = -0.02, *p* = .89, FSIQ, r(64) = 0.17, *p* = .17, episodic memory, r(65) = 0.14, *p* = .25, or executive function, r(67) = 0.14, *p* = .25, or global cognition, r(66) = 0.23, *p* = .10. There was no group effect of gender on SCD composite, t(66) = -0.34, *p* = .74.

### Influence of Error Awareness on SCD Composite and Domains

**Fig 1:**
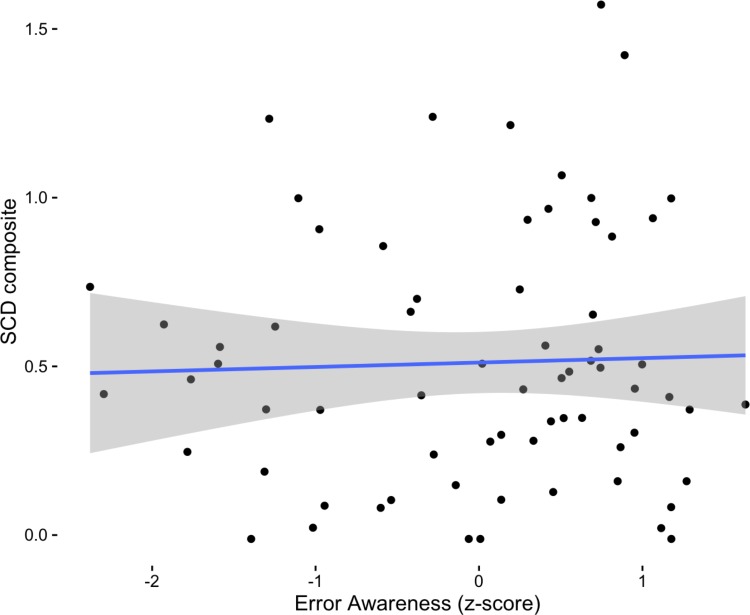
Association between SCD composite and level of error awareness on the EAT task (as represented by a z-score).

**Fig 2:**
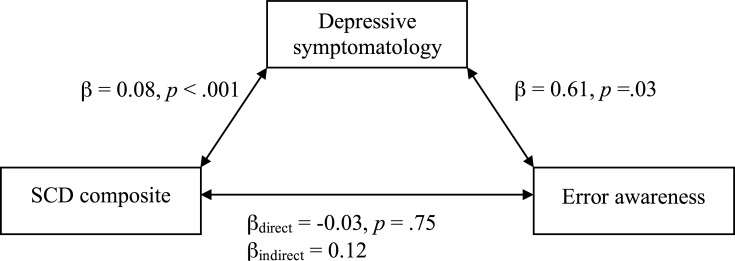
Mediation models of the direct and indirect effects of depressive symptoms on the relationship between SCD and error awareness.

Error awareness was not found to be associated with the SCD composite score, r(67) = 0.08, *p* = .52 (see Fig 1), or other SCD domains. A hierarchical regression analysis suggested that GDS (β = 0.47, *p* < .001) was the sole predictor of SCD in a model including for age, education, episodic memory, executive function, and error awareness (R^2^_full model_ = 49%, *F*_*change*_ = 0.73, *p* = .40). A post-hoc mediation analysis found a significant indirect effect of depressive symptoms on the relationship between error awareness and SCD (β = .14, 95%CI: 0.03, 0.29, see Fig 2). Further, a Sobel test showed full mediation in the model (*z* = 1.95, *p* = .05), suggesting that greater levels of depressive symptomatology highlighted a positive relationship between SCD and error awareness.

### Mediation Analysis: Indirect Effect of SCD on Objective Performance

**Fig 3:**
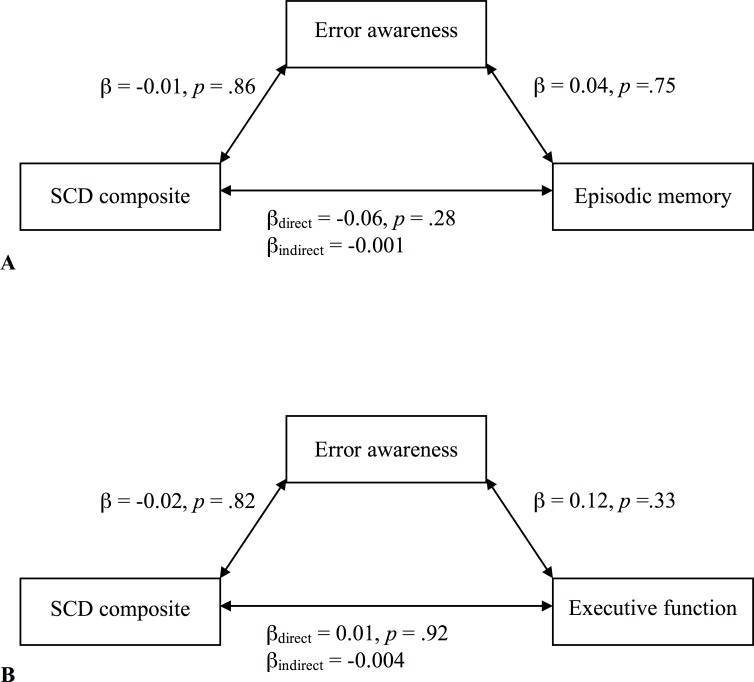
Mediation models of the direct and indirect effects of error awareness on the relationship between SCD. Episodic memory (A) and executive function (B).

Two mediation analyses were conducted to measure direct and indirect effects of error awareness on the relationship between SCD and objective performance. No indirect effects via error awareness were found between SCD and episodic memory, SCD and executive function, or SCD and global cognition. Fig 3A and 3B depict the relationships with episodic memory and executive function.

## Discussion

Error awareness was not found to contribute to the discrepancy between SCD and objective cognitive performance in cognitively normal older adults. While a large amount of individual variability in error awareness was found in our population, contrary to our expectations, we did not find this to covertly influence subjective cognitive concerns. Our findings further supported the well-established finding that depressive symptomatology, even at non-clinical levels, explain a significant proportion of variability in SCD in community-based older adults [3, 24–27].

Depressive symptomatology was also found to significantly influence error awareness, indicating a similar underlying influence of negative mood on SCD and awareness. While error awareness has not been investigated in relation to depression in older adults, it is possible that depressive symptomatology, even at non-clinical levels, has the potential to influence many higher-level cognitive processes in older adults [28]. To support this notion, in a post-hoc mediation analysis, we found an indirect relationship between error awareness and SCD as mediated by level of depressive symptomatology. That is, with greater levels of depressive symptomatology, a stronger positive relationship existed between SCD and error awareness. One possible rationale for this mediation is that depression amplifies the metacognitive monitoring of negative occurrences in the individuals’ environment, such as hyper-vigilance for errors. Self-focus has been found to relate to negative affect, specifically with regard to private or inward focus on one’s goals, thoughts and feelings (for a meta-analysis, see [29]). The experience of negative affect might increase an individuals’ tendency to engage in rumination, which may well lead to increased salience of subjective concerns and error awareness.

Contrary to our hypothesis, error awareness was not found to mediate the relationship between subjective concerns and cognitive performance. The rationale behind this prediction was that awareness of everyday errors might best represent the subjective observation of everyday memory failures [5], and so mediate the obfuscated relationship with objective cognitive performance. As our findings are at odds with Harty and colleagues [5] that suggested error awareness in older adults mediated the discrepancy between self-reported attentional control and memory performance, it would be inappropriate to conclude that there is no role for error awareness to play, but simply to say that the story may not be as clear as was previously predicted. The previous study utilized the same error awareness task, and found that older adults underreported error lapses in comparison with younger adults, and that error awareness was specifically related to objective attentional deficits. Some major methodological differences exist between our two studies; Harty and colleagues [5] measured subjective-objective discrepancy (which arguably deals with changing insight), while our study focused on the use of a widely-validated measure of SCD which focuses on subjective cognitive change. As such, it is possible declining insight may be related to attentional deficits in error awareness, but that subjective cognitive change, unlike insight, is only indirectly related to error awareness via depressive symptoms.

This study was conducted in cognitively-normal older adults who have not been investigated in relation to Alzheimer’s disease (AD) biomarkers, such as genetic risk, brain atrophy, or neocortical amyloid burden, which have all been shown to influence SCD. As such, these results cannot be generalised in the context of the preclinical stages of AD. Furthermore, there were no measures of insight or informant concerns included in this study, which could add further information about other factors that might mediate the relationship between SCD and objective cognitive performance. As many previous studies have shown, the influence of depressive symptomatology on subjective, metacognitive monitoring and self-reflective measures cannot be discounted [3, 25, 30]. It is possible that early signs of both depressive symptoms and greater SCD in cognitively-normal older adults may well be indicative of overall ‘illness’ due to a milieu of disease-related effects [31], and thus represent greater risk of progression to dementia. The current study cannot interrogate this question directly; it can, however, provide support to the notion that depressive symptoms amplify self-relevant errors and self-endorsed cognitive change.

## Supporting Information

S1 Fig Scatterplot of SCD composite in relation to Geriatric Depression Scale score. (TIFF)

S2 Fig Scatterplot of error awareness in relation to Geriatric Depression Scale score. (TIFF)
