# Supplementary figures and images for: Assessing Error Awareness as a Mediator of the Relationship between Subjective Concerns and Cognitive Performance in Older Adults
Source: PLoS One. 2016 Nov 10;11(11):e0166315. doi: 10.1371/journal.pone.0166315 (PMC5104449; doi:10.1371/journal.pone.0166315)

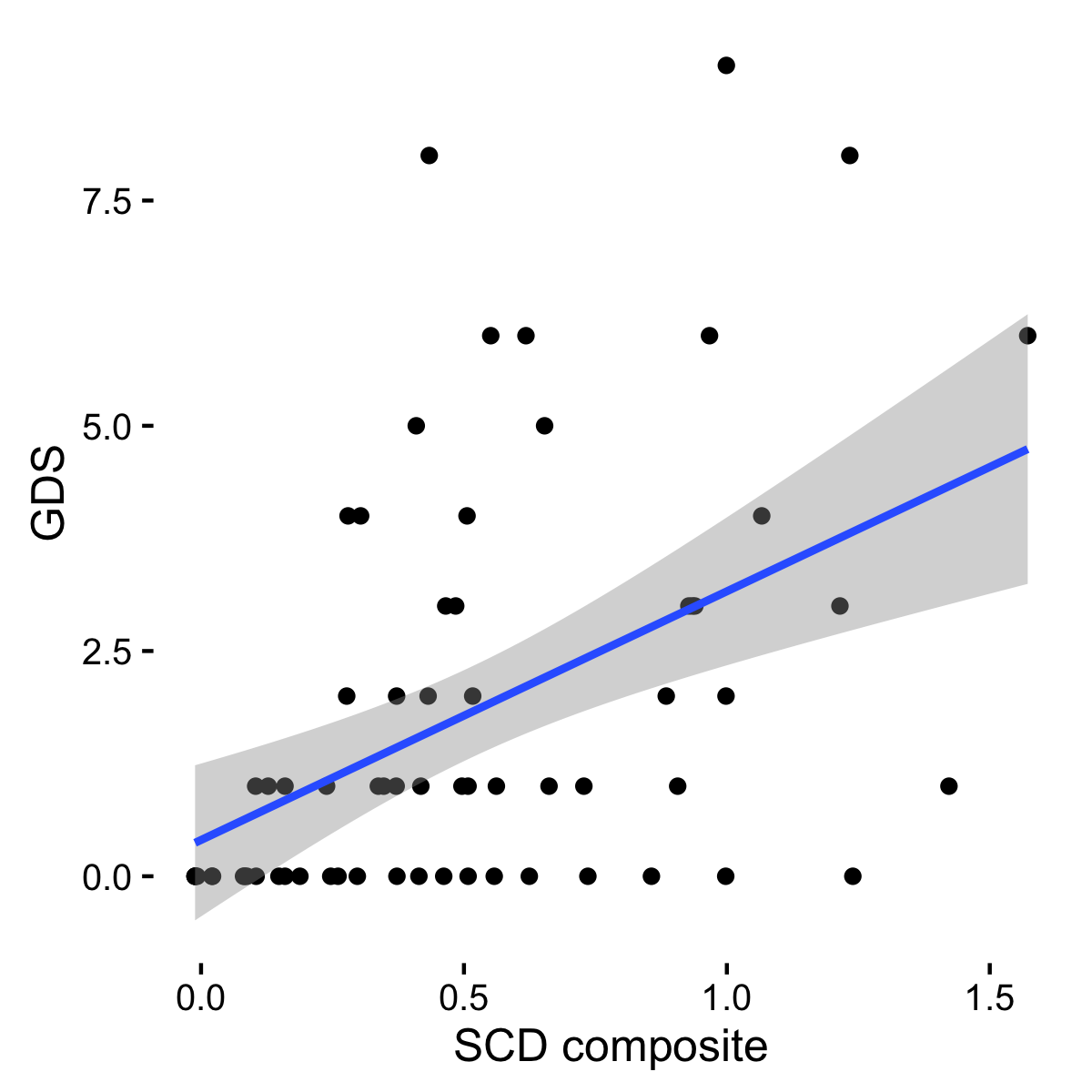

Supplement: S1 Fig — (TIFF) [file pone.0166315.s001.tiff]

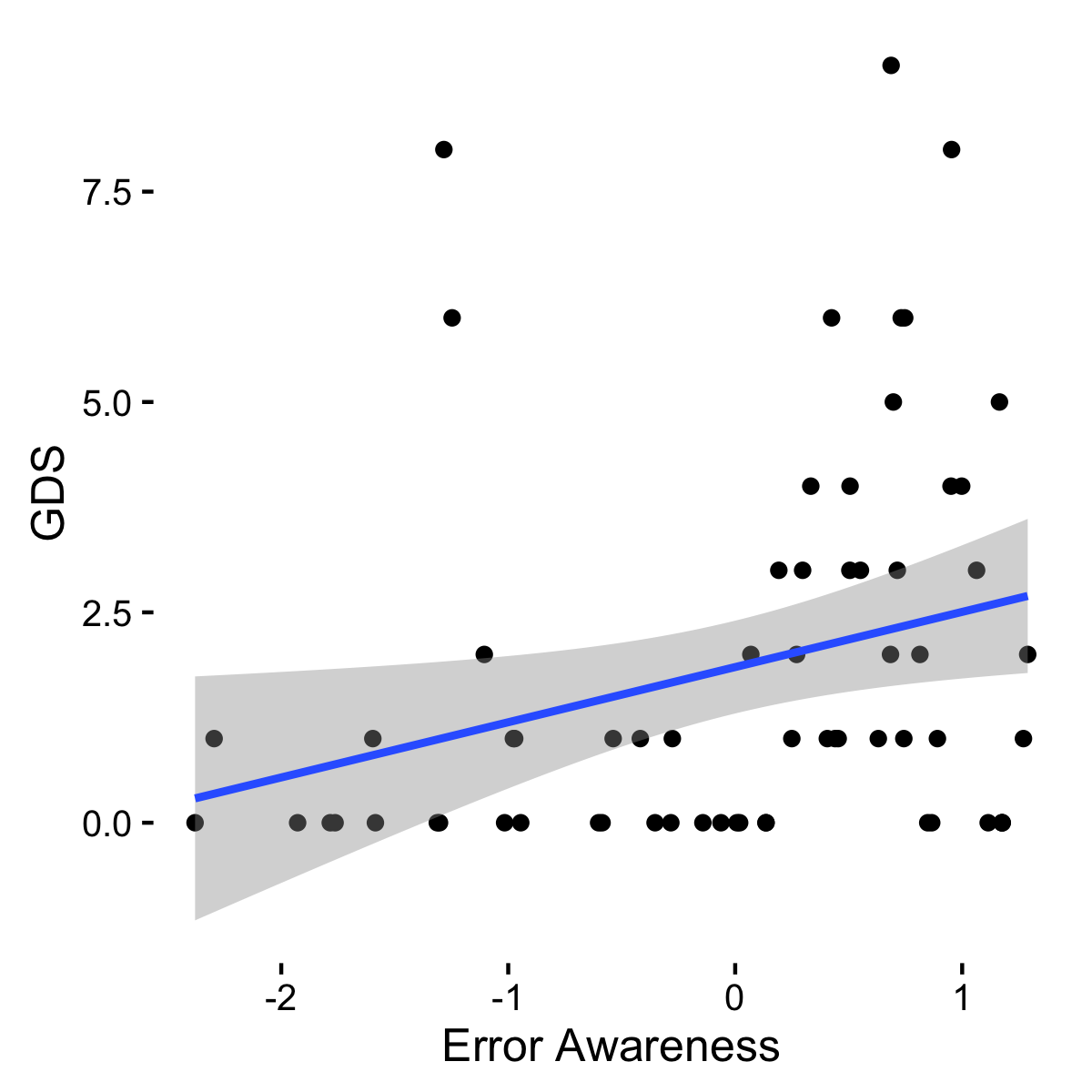

Supplement: S2 Fig — (TIFF) [file pone.0166315.s002.tiff]
